# A systematic review and meta-analysis of prognostic biomarkers in resectable esophageal adenocarcinomas

**DOI:** 10.1038/s41598-018-31548-6

**Published:** 2018-09-05

**Authors:** Aafke Creemers, Eva A. Ebbing, Thomas C. Pelgrim, Sjoerd M. Lagarde, Faridi S. van Etten-Jamaludin, Mark I. van Berge Henegouwen, Maarten C. C. M. Hulshof, Kausilia K. Krishnadath, Sybren L. Meijer, Maarten F. Bijlsma, Martijn G. H. van Oijen, Hanneke W. M. van Laarhoven

**Affiliations:** 1 Laboratory of Experimental Oncology and Radiobiology, Amsterdam UMC, Univ of Amsterdam, Cancer Center Amsterdam, Amsterdam, The Netherlands; 2 Department of Medical Oncology, Amsterdam UMC, Univ of Amsterdam, Cancer Center Amsterdam, Amsterdam, The Netherlands; 3 Department of Surgery, Erasmus Medical Center, Rotterdam, The Netherlands; 4 Department of Medical Library Science, Amsterdam UMC, Univ of Amsterdam, Cancer Center Amsterdam, Amsterdam, The Netherlands; 5 Department of Surgery Amsterdam UMC, Univ of Amsterdam, Cancer Center Amsterdam, Amsterdam, The Netherlands; 6 Department of Radiotherapy, Amsterdam UMC, Univ of Amsterdam, Cancer Center Amsterdam, Amsterdam, The Netherlands; 7 Department of Gastroenterology, Amsterdam UMC, Univ of Amsterdam, Cancer Center Amsterdam, Amsterdam, The Netherlands; 8 Department of Pathology, Amsterdam UMC, Univ of Amsterdam, Cancer Center Amsterdam, Amsterdam, The Netherlands

## Abstract

Targeted therapy is lagging behind in esophageal adenocarcinoma (EAC). To guide the development of new treatment strategies, we provide an overview of the prognostic biomarkers in resectable EAC treated with curative intent. The Medline, Cochrane and EMBASE databases were systematically searched, focusing on overall survival (OS). The quality of the studies was assessed using a scoring system ranging from 0–7 points based on modified REMARK criteria. To evaluate all identified prognostic biomarkers, the hallmarks of cancer were adapted to fit all biomarkers based on their biological function in EAC, resulting in the features angiogenesis, cell adhesion and extra-cellular matrix remodeling, cell cycle, immune, invasion and metastasis, proliferation, and self-renewal. Pooled hazard ratios (HR) and 95% confidence intervals (CI) were derived by random effects meta-analyses performed on each hallmarks of cancer feature. Of the 3298 unique articles identified, 84 were included, with a mean quality of 5.9 points (range 3.5–7). The hallmarks of cancer feature ‘immune’ was most significantly associated with worse OS (HR 1.88, (95%CI 1.20–2.93)). Of the 82 unique prognostic biomarkers identified, meta-analyses showed prominent biomarkers, including COX-2, PAK-1, p14ARF, PD-L1, MET, LC3B, IGFBP7 and LGR5, associated to each hallmark of cancer.

## Introduction

Esophageal carcinomas can be divided into two distinct histological subtypes; squamous cell carcinoma (ESC) and adenocarcinoma (EAC). In Northwestern European countries and North America a rapid rise in the incidence of EAC is seen^1,2^. Mainly due to late symptoms, only half of the patients present with curable disease and despite multimodality treatment, median overall survival remains merely 48.6 months in patients with operable disease^3^.

To increase survival, biomarkers could harbor great potential by (i) better stratification of patients according to their tumor biology and (ii) to direct the development of new targeted anti-cancer therapies. Prognostic biomarkers provide information on clinical cancer outcomes, such as overall survival (OS), independent of received treatment^4^. The Erb-b2 receptor tyrosine kinase 2 (Neu or HER2), a member of the epithelial growth factor receptor family, has previously been identified as such a prognostic biomarker in EAC, which can be targeted by trastuzumab, a humanized anti-HER2 monoclonal antibody^5^. Since a significant survival benefit was shown in the phase III ToGA trial, trastuzumab in addition to standard chemotherapy, has become standard of care for HER2 positive advanced-stage gastro-esophageal cancers^5,6^. Currently, the value of HER2 directed therapies in patients with curative EAC is investigated (NCT02120911), however, compared to other tumor types, targeted therapy development is lagging behind in EAC. Thus far, trastuzumab is the only available targeted treatment option in EAC, while survival in this disease remains dismal, underscoring the urgent need to improve therapeutic options^7^. Further identification of prognostic biomarkers may lead to the development of new targeted therapies, thereby improving survival.

Unfortunately, previous reviews investigating prognostic biomarkers in esophageal cancer did not distinguish EAC from ESC or solely focused on immunohistochemistry (IHC) as the method of biomarker detection^8,9^. However, great differences in tumor biology between EAC and ESC have been demonstrated, necessitating separate analysis^2^. Furthermore, since their publication there has been an enormous development of detection techniques, enhancing the opportunity to identify clinically applicable prognostic biomarkers^10^. And lastly, the REporting recommendations for tumor MARKer prognostic studies (REMARK criteria) have become consensus guidelines for prognostic biomarker studies, to increase quality of the published work and improve extrapolation of the study outcomes^11^. Hence, when appraising new prognostic biomarkers, these REMARK criteria should be taken into account.

This systematic review with meta-analyses provides an overview of the prognostic biomarkers in resectable EAC treated with curative intent, focusing on overall survival, to guide the development of new targeted therapies.

## Results

### Study characteristics

**Figure 1:**
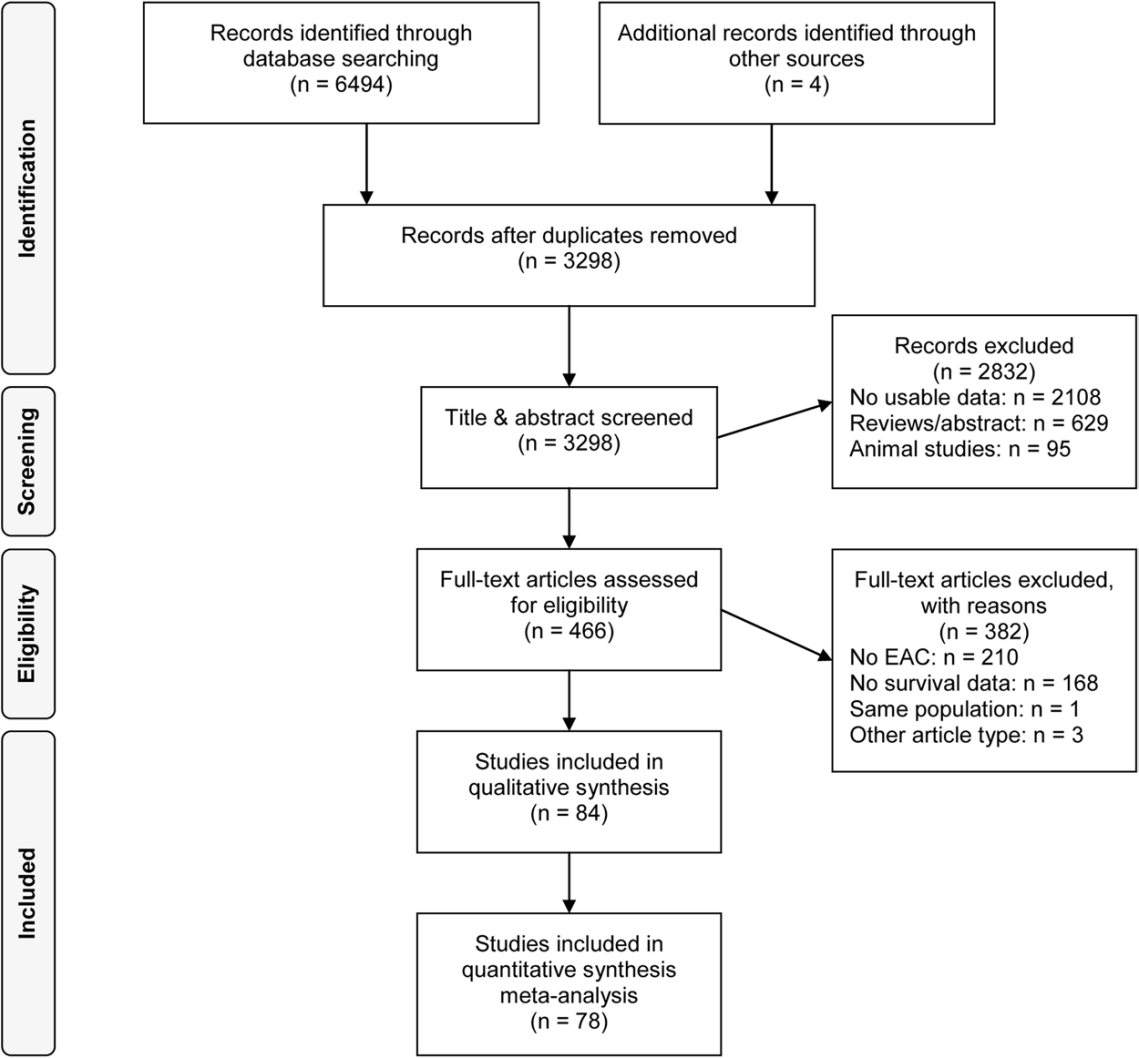
Flow-chart of included articles.

All 3,298 identified articles were screened on title and abstract (Fig. 1). After assessing 466 articles on full text, 84 articles were included^12–95^. Six articles were grouped in the adapted hallmark of cancer ‘multiple’, resulting in 78 articles that could be included in the meta-analysis, investigating a total population of 12,876 EAC patients. The main characteristics of the studies are shown in supplementary Table S1. A total of 82 unique biomarkers were identified. The majority of the biomarkers were detected by immunohistochemistry (IHC) or a combination of IHC and an *in situ* hybridization method (ISH). Less frequently applied detection methods were PCR, RNA sequencing, DNA sequencing and one article used a combination of reverse phase protein array (RPPA) analysis, reverse transcriptase-PCR and IHC^95^. Most (N = 61) articles included a study population consisting of EAC only, 12 articles included an EAC population that consisted of ≥70% adenocarcinomas, 11 articles performed separate OS analyses on EAC and other histological subtypes. Of the assessed patients, 1822 (14.2%) received prior chemo(radiation)therapy. The mean study sample size and IF of the articles was 152 patients (standard deviation = 112.16) and 4.54, respectively.

### Quality assessment

Assessment of the study quality using the adapted REMARK criteria, resulted in a mean quality of 5.9 points (range 3.5–7) (Supplementary Table S2). Three studies had a low quality score, and were included in the sensitivity analyses^31^. In general, points were lacking in quality criteria C5; reporting if patients received therapy and if so, specifying the chemo(radio)therapy regimen. In addition, C1; a representative cohort with clear baseline characteristic and C2; reasons of patient drop-out, were often absent. A positive correlation (R = 0.480) was observed comparing study size and the impact factor of the journal in which the study was published (p = 0.0005) (Supplementary Fig. S3). There was no correlation (R = 0.058) between the study quality assessed by the adapted REMARK criteria and impact factor (p = 0.601).

### Proliferation

**Figure 2:**
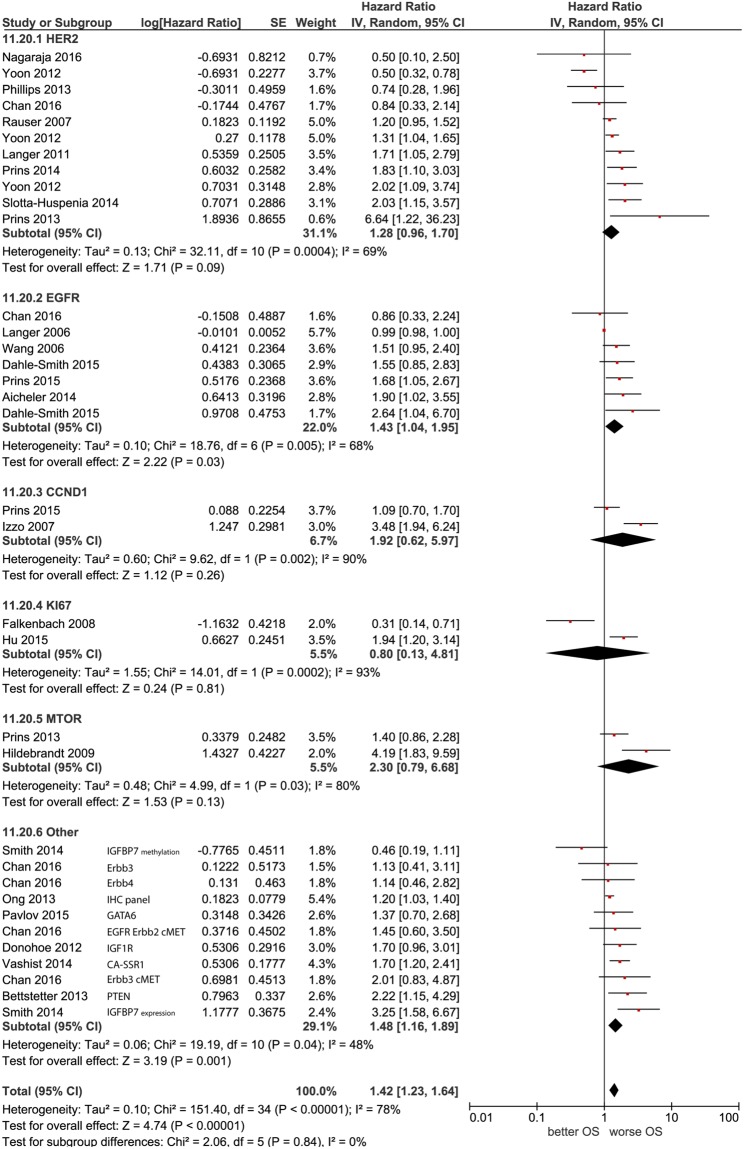
Random-effect Forest plot of prognostic biomarkers included in the adapted hallmark of cancer ‘proliferation’. EGFR, Cyclin D1, mTOR and HER2 were pooled as subgroup.

The majority of the biomarkers studied are involved in tumor cell proliferation, of which HER2, EGFR, cyclin D, KI67 and MTOR were the most frequently reported (Fig. 2). Subgroup analysis on EGFR demonstrated an association with worse OS, HR 1.43 (95% CI 1.04–1.95). Analyses of the HER2 subgroup, however, showed no significant association with OS, HR 1.28 (95% CI 0.96–1.70). HER2 remained not significantly associated with worse OS when evaluating the HER2 subgroup by including only data on HER2 expression assessed by means of the gold standard (IHC and in case of equivocal HER2 expression (Hoffman scoring system 2+) an additional *in situ* hybridization method^96^), or if data on EAC with Barrett’s esophagus (BE) segment was replaced by data on EAC without BE ((HR 1.09 (95%CI 0.46–2.60)) and (HR 1.33 (95%CI 0.78–2.28)), respectively) (Table 1). The overall pooled effect of the proliferation feature was significantly associated with worse OS (HR 1.41 (95%CI 1.22–1.63)), however, significant test heterogeneity was found. IGFBP7, a member of the insulin like growth factor receptor family, was identified as most promising prognostic biomarker in this hallmarks of cancer feature. Funnel plot analyses showed no indication for publication bias (Supplementary Material S4).

**Table 1:** Sensitivity analyses on the HER2 subgroup.

HER2 subgroup analyses	HR (95% CI)	p-value	Population
HER2 total	1.28 (0.96–1.70)	0.09	2225
HER2 IHC/ISH only	1.09 (0.46–2.60)	0.84	1232
HER2 IHC/ISH only; without BE	1.33 (0.78–2.28)	0.30	1232

### Hallmark specific markers

**Figure 3:**
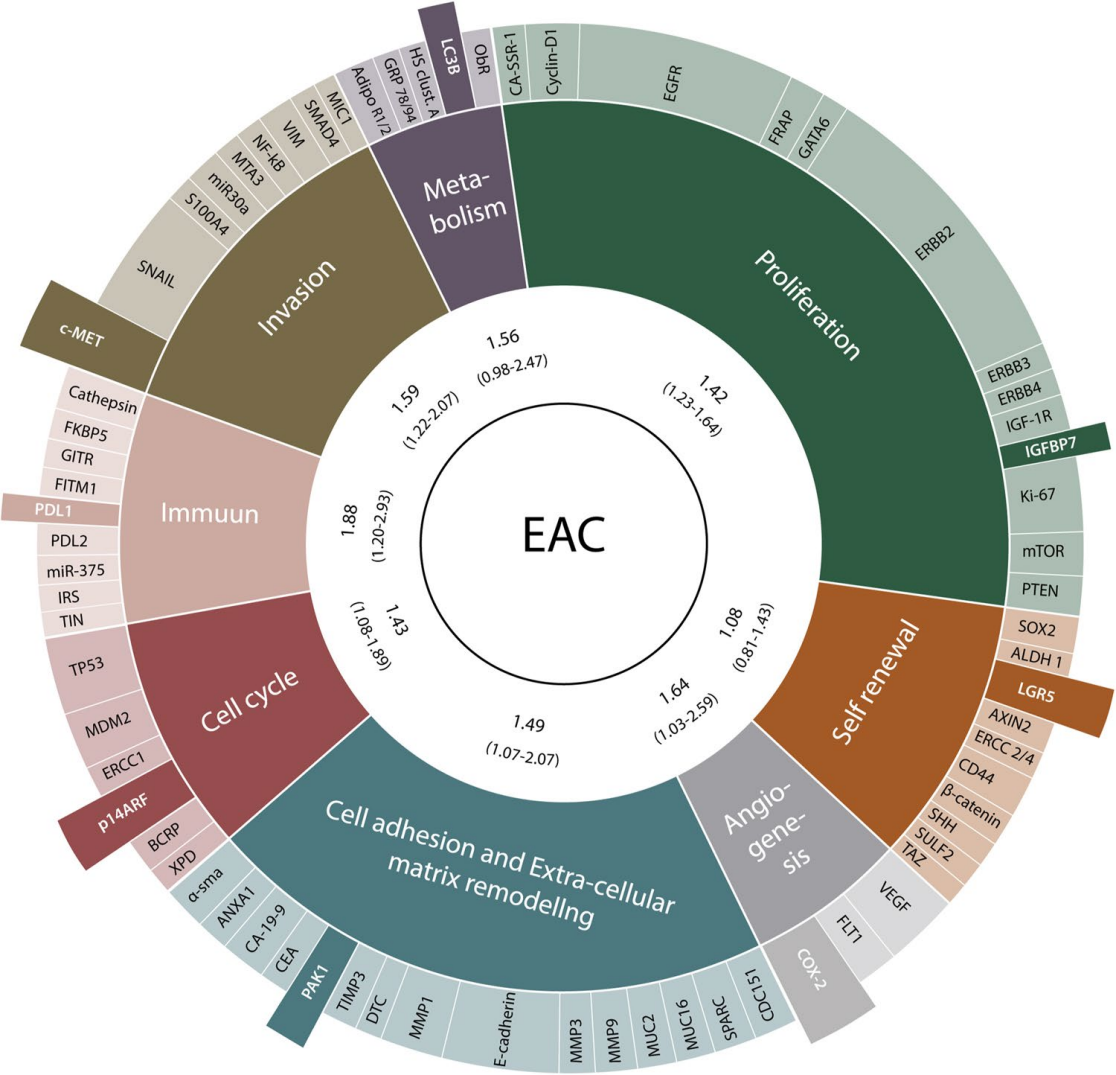
All identified biomarkers and adapted hallmarks of cancer are summarized in the Ferris Wheel Plot. The area of each adapted hallmark of cancer represents the amount of articles with data on the corresponding hallmark of cancer. The most promising prognostic biomarkers according to our meta-analysis are highlighted. In the inner circle the hazard ratios (HR) and 95% Confidence Intervals (95%CI) are reported for each adapted hallmark of cancer.

All identified biomarkers and hallmarks of cancer features are summarized in Fig. 3. The potential of all identified prognostic biomarkers was evaluated by assembling the biomarkers according to their main function in tumor biology in their corresponding hallmarks of cancer feature. Performing meta-analysis on all features, most were significantly associated with worse OS, except metabolism (HR 1.56 (95%CI 0.98–2.47)), and self-renewal (HR 1.08, (95%CI 0.81–1.43)). The hallmark of cancer feature ‘immune’ was most significantly associated with worse OS (HR 1.88, (95%CI 1.20–2.93)). Of the 82 unique prognostic biomarkers identified, meta-analyses showed several promising biomarkers, including COX-2, PAK-1, p14ARF, PD-L1, MET, LC3B and LGR5, associated to each hallmark of cancer feature. After excluding low study quality articles, there was no significant association with OS in the group cell adhesion (N = 1, n = 52, SPARC and SPP1; HR 1.49 (95% CI 1.07–2.07) to HR 1.24 (95% CI 0.83–1.86), respectively) (Table 2)^31,45,58^. Additional sensitivity analyses on EAC treated with surgery as single treatment modality vs. EAC treated with neoadjuvant treatment and surgery, the hallmarks of cancers feature ‘cell cycle’ was not significantly associated with OS (HR 1.43 (95%CI 1.08–1.89) to HR 1.09 (95%CI 0.75–1.57), respectively) although the same biomarkers were tested. The feature ‘metabolism’ remained not significantly associated with OS. After sensitivity analyses, the prognostic biomarkers identified as most promising remained unchanged for each hallmark of cancer feature. Funnel plot analyses showed no indication for publication bias.

**Table 2:** Sensitivity analysis on articles with a low quality score on the adapted REMARK criteria and those patients receiving (neo)adjuvant chemotherapy.

Ferris wheel plot subgroup analyses	HR (95% CI)	p-value
**REMARK ≤ 3**.**5**		
Cell adhesion (Kim 2010)	1.24 (0.83–1.86)	0.30
Immuun (Derks 2015)	2.18 (1.34–3.55)	0.0002
Proliferation (Vashist 2014)	1.41 (1.22–1.63)	0.000
**Neoadjuvant chemo(radio)therapy**		
Cell cycle (Bradburry 2009)	1.09 (0.75–1.57)	0.65
Metabolism (El-Mashed 2015)	1.34 (0.93–1.92)	0.12

## Discussion

This review summarizes the great diversity of prognostic biomarkers studied in EAC thus far. Evaluating the biomarkers by grouping them based on their role in tumor biology to the most fitted hallmark of cancer feature, 82 unique biomarkers could be identified.

Interestingly, the hallmark of cancer feature ‘immune’ presented itself as most significant associated with worse OS, and therefore may harbor potential to apply targeted therapies. Due to increased understanding of the tumor immunomicro-environment, and promising trial results, new immune based therapies are recently emerging, such as the PD-L1/PD1 targeting agents nivolumab and pembrolizumab^97^. Targeting PD-L1/PD-1, a critical immune checkpoint, releases the inhibitory effect on both the humoral and cellular immune response, activating T-cells to enhance the antitumor response. These PD-1 pathway inhibitors have previously been FDA approved in several solid tumors, including melanoma and non-small lung cancer. Indeed here we identify PD-L1, a ligand of the co-inhibitory receptor PD-1, as the most promising prognostic biomarker included in this hallmarks of cancer feature. However, the clinical applicability of these drugs has not been proven in resectable EAC yet and whether PD-1 is a predictive biomarker, reflective of response to treatment, remains to be elucidated^4,97^.

For all other hallmarks of cancer features promising prognostic biomarkers were identified as well, including COX-2, PAK-1, p14ARF, MET, LC3B, IGFBP7 and LGR5. For the MET-, IGFBP7, and LGR5 pathways targeted therapies have already been studied in other cancer types with varying results, however, the potential to target these biomarkers in EAC is yet to be investigated^98,99^. Likewise, the inhibition of CDK4/6 in p14ARF mutant patients by small molecules or pan-CDK inhibitors is being invested as add-on to standard chemotherapy backbones, potentially enabling blockage of unrestricted cell division caused by p14ARF mutations^100^. Non-steroidal anti-inflammatory drugs (NSAID’s), inhibiting COX-2, are commonly used and safe. Hence, inhibition of COX-2, an important regulator of cell growth, differentiation and apoptosis, may be a valuable contribution in the treatment of EAC. Thus far, COX-2 has been demonstrated to be involved in the neoplastic formation of esophageal cancer^101^. Moreover, the use of NSAID’s, is associated with a reduced risk of EAC development and is proven to reduce cell growth in 8 esophageal cell lines. Contrary, yet little is known about the potential drugability of PAK-1 in cancer, even though the recently elucidated central role in oncogenic signaling has enhanced interest in small-molecule based PAK-1 targeting^102^. Similarly, merely *in vitro* the inhibition of autophagy by blocking LC3B has been explored in oncological diseases. Therefore, the therapeutic potential remains to be clarified.

Even though promising prognostic biomarkers were identified, limitations should be recognized. Firstly, after performing sensitivity analysis on the study quality, the feature cell adhesion was no longer significantly associated with OS when excluding articles scoring low on the adapted REMARK criteria^31,45,58^. In addition, as it is known that studies with low quality hamper extrapolation of the data to clinical practice, it is surprising to notice that study size and impact factor were correlated, while no correlation between the study quality and the impact factor was found. Although after sensitivity analyses on articles scoring low on the adapted REMARK criteria the same promising biomarkers were still identified, the varying study quality is worrying. Frequently, articles failed to report the received therapy, and if this information was supplied, often did not specify the treatment regimen. As nowadays neoadjuvant treatment has become standard of care for operable EAC, reporting these baseline characteristics has become increasingly important.

In this meta-analyses 1822 (14.2%) resection specimens were evaluated on prognostic biomarker status after patients received neoadjuvant chemo(radiation)therapy. It should be noted that in specimens of good-responders no, or a few, remaining tumor cells may be found, biasing the prognostic potential of the assessed biomarker. Moreover, if post-neoadjuvant therapy samples are included in biomarker analyses, treatment regimens should be clearly described. It is known that a better response to therapy is attained with neoadjuvant chemoradiation therapy than if patients receive radiation therapy as single treatment modality. This could further bias the results found. In addition, when extrapolating these results to a predictive setting for the identification of new therapy options, these biomarkers might not have predictive potential in the neoadjuvant setting. Indeed, sensitivity analyses on articles reporting on patients who received neoadjuvant therapy demonstrated the influence of these treatment regimens on the association between biomarker status and survival. The feature ‘cell cycle’ was significantly associated with worse OS in all patients, and, when testing the same biomarkers, no longer harbored this association with survival if solely neoadjuvant treated EAC was included in the analysis. Since commonly used DNA-damaging chemotherapeutics as carboplatin and paclitaxel have influence on the cell cycle, this effect was expected, highlighting the importance of reporting the received treatment regimen.

The importance of clear reporting standards for biomarker research and standardization of the detection method used is also demonstrated by subgroup analyses on HER2. In contrast to the current notion, no association with decreased survival was found when plotting the data of all articles reporting on the prognostic potential of HER2. When exclusively including data on HER2 positivity assessed by means of the gold standard, IHC and in case of equivocal HER2 expression (Hoffman scoring system 2+) an additional *in situ* hybridization method, the association with worse OS remained not significant^5,103^. The significant test heterogeneity found in the corresponding hallmark of cancer feature ‘proliferation’ could at least partly be attributed to the varying detection methods applied. As all used tests have a unique sensitivity and specificity, outcomes can be greatly influenced by the method of biomarker assessment. The applied detection method will not only reflect underlying tumor biology, but also affect the relation of the biomarker with prognostic outcomes and targetability. For example, it has been demonstrated that solely assessing HER2 positivity by amplification of the HER2-gene with an *in situ* hybridization method does not correlate to efficacy of HER2-targeted therapy^103^. Likewise, different IHC cutoff-points of biomarker positivity influence both prognostic and predictive outcomes. As has been demonstrated in this meta-analysis, even for well-known biomarkers such as HER2, used in clinical practice, articles use varying definitions of biomarker positivity, thereby limiting comparison of data. Several promising biomarkers in resectable EAC have been identified, however, in order to stratify patients in accordance to their tumor biology, and to develop new targeted anti-cancer treatments, future research is needed. First, standardization of reporting on biomarker research is needed to further identify prognostic biomarkers. Subsequently, large-scale multicenter randomized-controlled trials should be conducted to validate the clinical applicability of these biomarkers and to evaluate their potential targetability.

To conclude, a wide variety of prognostic proteins and their expression have been studied in EAC treated with curative intent. Despite varying study quality of the published data, promising biomarkers could be identified, including COX-2, PAK-1, p14ARF, PD-L1, MET, LC3B, IGFBP7 and LGR5. The clinical application and targetability of these biomarkers as anti-cancer therapy in operable EAC should be addressed in future research.

## Methods

### Search strategy

Literature was retrieved using the Medline, Cochrane and Embase databases on the 19^th^ of January 2017 to identify articles published in the last 10 years, with the publication date restricted to the first of January 2007 until the first of January 2017. In addition to MESH terms, free text words were added to the search, to include all relevant articles that might not have assigned MESH terms yet. The full search is available in the supplementary information S5.

### Screening and selection of studies

All titles, abstracts and full text articles were screened independently by two researchers (AC and EAE), discrepancies were resolved by discussion. Articles were selected based on the following criteria; (i) the research population included adenocarcinomas of the esophagus or the gastro-esophageal junction, defined as Siewert class I and II, that could be treated with curative intent (ii) should report biomarker related overall survival (OS) data, described with hazard ratios (HR), 95% confidence intervals (CI), and *p*-value. If both EAC and ESC were studied, the research population should include at least 70% EAC or display separate survival analysis. Reviews, case reports, (meeting) abstracts, phase I studies and articles without full-text in English were excluded. When articles reported on the same biomarker(s) investigating the identical patient population, the publication examining the most biomarkers was included. Endnote X7 (Clarivate Analytics, Boston, USA) was used to select and screen the literature.

### Data extraction and outcomes

Data extraction was done by AC and EAE following a predefined protocol and double checked until consensus was reached. The following data was extracted: first author, publication year, journal, patient population (EAC only, >70% EAC or EAC and ESC with separate survival analysis), tumor material studied (blood, biopsy, resection specimen or a combination), reported tissue handling, method of biomarker detection, used scoring methods and cut-off values for biomarker positivity, received therapy (yes (including a clear description of the treatment regimen), no, or not reported (NR)), the duration of follow-up, and reported confounders in multivariate analyses. Lastly, the primary outcome of this review, overall survival data of univariate and/or multivariate analyses presented as HR, 95% CI, and *p*-value. The impact factor (IF) of journals at the time of publication of the studies were extracted from bioxbio.com/if/.

### Study quality assessment

To assess the quality of the included studies the REporting recommendations for tumor MARKer prognostic studies (REMARK) criteria for biomarker studies were adapted into a scoring system (Table 3)^11^. The adapted scoring criteria were chosen by discussion between AC, EAE, MvO and HvL. The articles could be scored 1 point per item, with a maximum of 7 points. In case of ambiguity or incompleteness, half a point was allocated. A study was defined of low quality when ≤3.5 points were assigned. The study quality was assessed by AC and EAE, in case of disagreement consensus was reached by discussion.

**Table 3:** The adapted version of the REporting recommendations for tumor MARKer prognostic studies (REMARK) criteria for biomarker studies^11^.

Adapted REMARK criteria for Quality Assessment (1 point/criteria)
**1**. Case selection adequate and representative (baselines form medical chart, including TNM, differentiation and location) (=1 point)
**2**. Clear description of the flow of patients through the study and reasons of dropout (=1 point)
**3**. Used tumor material (biopsy/ resection specimen or both) (=1 point)
**4**. Reporting the tissue handling, method of biomarker detection and clear description of scoring criteria (=1 point)
**5**. If patients received therapy, a clear description of the treatment regimen: reported (=1 point), if treatment is unspecified (=1/2 point)
**6**. The duration of follow-up (=1 point)
**7**. A clear description of the confounders used in multivariate analyses (=1 point, ½ point if only univariate analyses are conducted)

A study could be allocated one point for each of the seven criteria, in case of ambiguity, half a point was assigned. Sensitivity analyses were performed on studies assigned ≤3,5 points on the adapted REMARK criteria scale.

### Statistics

The potential of all identified prognostic biomarkers was evaluated by grouping the biomarkers according to their main function in tumor biology in the corresponding hallmark of cancer^104^. To fit all identified biomarkers, the hallmarks of cancer were adapted, resulting in the following features: angiogenesis, cell adhesion and extra-cellular matrix remodeling, cell cycle, immune, invasion and metastasis, metabolism, proliferation, and self-renewal. Some articles showed data on a cluster of genes, these were assembled in the hallmarks of cancer feature ‘multiple’. Due to the heterogeneous scope of action of the biomarkers, we did not perform meta-analysis on papers included in the ‘multiple’ group. Pooled hazard ratios (HR) and 95% confidence intervals (CI) were derived by random effects meta-analyses performed on each hallmark of cancer feature. HR and 95%CI data of univariate and multivariate analysis were combined in the meta-analysis; data derived from multivariate analysis was used as default, but when absent, univariate values were used. If the data was related to absence rather than presence of the biomarker, the HR data were inversed. When identical biomarkers were reported in more than two studies, these duplicate biomarkers were included in subgroup analysis. In order to determine the influence of a low quality score, sensitivity analyses were performed on studies with a low study quality on the adapted REMARK criteria scale. Additional sensitivity analyses were conducted on studies showing data on both EAC treated with surgery as single treatment modality and neoadjuvant treated EAC. Finally, the most promising biomarker for each hallmark of cancer feature was selected based on the most optimal combination of a high HR and small 95% CI. Consensus was reached between AC, EAE, MvO and HvL on the selected biomarkers. Publication bias was evaluated by means of a Funnel plot on all hallmarks of cancer features. Random effects meta-analyses were performed in Review Manager V5 (The Cochrane Collaboration, Copenhagen, Denmark). Pearson’s correlations with linear regression analysis between IF, adapted REMARK quality score, and patient cohort size were performed using GraphPad Prism 6 (GraphPad Software, La Jolla, CA, USA).

### Ethics statement

This article does not contain any studies with human or animal subjects performed by any of the authors.

## Electronic supplementary material

Supplementary Information S2-S5

Supplementary Information table S1

## References

[1] Rubenstein JH, Shaheen NJ (2015). Epidemiology, diagnosis, and management of esophageal adenocarcinoma. Gastroenterology.

[2] Rustgi AK, El-Serag HB (2014). Esophageal carcinoma. New England Journal of Medicine.

[3] Shapiro, J. *et al*. Neoadjuvant chemoradiotherapy plus surgery versus surgery alone for oesophageal or junctional cancer (CROSS): long-term results of a randomised controlled trial. *The lancet oncology* **16**, 1090–1098 (2015). 10.1016/S1470-2045(15)00040-6 26254683

[4] Ballman KV (2015). Biomarker: predictive or prognostic?. Journal of Clinical Oncology.

[5] Bartley, A. N. *et al*. HER2 Testing and Clinical Decision Making in Gastroesophageal Adenocarcinoma: Guideline From the College of American Pathologists, American Society for Clinical Pathology, and the American Society of Clinical Oncology. *Journal of Clinical Oncology*, JCO. 2016.2069. 4836 (2016). 10.1200/JCO.2016.69.4836 28129524

[6] Bang Y-J (2010). Trastuzumab in combination with chemotherapy versus chemotherapy alone for treatment of HER2-positive advanced gastric or gastro-oesophageal junction cancer (ToGA): a phase 3, open-label, randomised controlled trial. The Lancet.

[7] Hosoda K, Yamashita K, Katada N, Watanabe M (2015). Overview of multimodal therapy for adenocarcinoma of the esophagogastric junction. General thoracic and cardiovascular surgery.

[8] Matthews LM (2015). Systematic review and meta-analysis of immunohistochemical prognostic biomarkers in resected oesophageal adenocarcinoma. British journal of cancer.

[9] Chen M, Huang J, Zhu Z, Zhang J, Li K (2013). Systematic review and meta-analysis of tumor biomarkers in predicting prognosis in esophageal cancer. BMC cancer.

[10] Metzker, M. L. Vol. 11.1 (2010), 31–46, (Nature reviews genetics, 2010). 10.1038/nrg2626 19997069

[11] McShane LM (2005). Reporting recommendations for tumor marker prognostic studies (REMARK). Journal of the National Cancer Institute.

[12] Bhandari P (2006). Prognostic significance of cyclooxygenase-2 (COX-2) expression in patients with surgically resectable adenocarcinoma of the oesophagus. BMC Cancer.

[13] Prins MJ, Verhage RJ, ten Kate FJ, van Hillegersberg R (2012). Cyclooxygenase isoenzyme-2 and vascular endothelial growth factor are associated with poor prognosis in esophageal adenocarcinoma. J Gastrointest Surg.

[14] Eng L (2015). Discovery and validation of vascular endothelial growth factor (VEGF) pathway polymorphisms in esophageal adenocarcinoma outcome. Carcinogenesis.

[15] Bradbury PA (2009). Vascular endothelial growth factor polymorphisms and esophageal cancer prognosis. Clinical Cancer Research.

[16] Xie LX (2013). Lymphangiogenesis and prognostic significance of vascular endothelial growth factor C in gastro-oesophageal junction adenocarcinoma. Int J Exp Pathol.

[17] Underwood TJ (2015). Cancer-associated fibroblasts predict poor outcome and promote periostin-dependent invasion in oesophageal adenocarcinoma. J Pathol.

[18] Wang KL (2006). Expression of annexin A1 in esophageal and esophagogastric junction adenocarcinomas: association with poor outcome. Clinical cancer research: an official journal of the American Association for Cancer Research.

[19] Tokunaga R (2015). Carbohydrate antigen 19-9 is a useful prognostic marker in esophagogastric junction adenocarcinoma. Cancer Med.

[20] Fisher OM (2016). CD151 Gene and Protein Expression Provides Independent Prognostic Information for Patients with Adenocarcinoma of the Esophagus and Gastroesophageal Junction Treated by Esophagectomy. Ann Surg Oncol.

[21] Vashist YK (2012). Disseminated tumor cells in bone marrow and the natural course of resected esophageal cancer. Ann Surg.

[22] Dong H (2013). The metastasis-associated gene MTA3, a component of the Mi-2/NuRD transcriptional repression complex, predicts prognosis of gastroesophageal junction adenocarcinoma. PLoS ONE.

[23] Falkenback D, Nilbert M, Oberg S, Johansson J (2008). Prognostic value of cell adhesion in esophageal adenocarcinomas. Dis Esophagus.

[24] Prins MJ (2015). The role of biological markers of epithelial to mesenchymal transition in oesophageal adenocarcinoma, an immunohistochemical study. Journal of clinical pathology.

[25] Bradbury PA (2009). Matrix metalloproteinase 1, 3 and 12 polymorphisms and esophageal adenocarcinoma risk and prognosis. Carcinogenesis.

[26] Lu X (2016). Evaluation of MMP-9 and MMP-2 and their suppressor TIMP-1 and TIMP-2 in adenocarcinoma of esophagogastric junction. Onco Targets Ther.

[27] Grimm M (2010). MMP-1 is a (pre-)invasive factor in Barrett-associated esophageal adenocarcinomas and is associated with positive lymph node status. Journal of translational medicine.

[28] Davison JM (2014). MUC2 expression is an adverse prognostic factor in superficial gastroesophageal adenocarcinomas. Human pathology.

[29] Streppel MM (2012). Mucin 16 (cancer antigen 125) expression in human tissues and cell lines and correlation with clinical outcome in adenocarcinomas of the pancreas, esophagus, stomach, and colon. Human pathology.

[30] Li Z (2015). Personalizing risk stratification by addition of PAK1 expression to TNM staging: improving the accuracy of clinical decision for gastroesophageal junction adenocarcinoma. Int J Cancer.

[31] Kim SM (2010). Prognostic biomarkers for esophageal adenocarcinoma identified by analysis of tumor transcriptome. PLoS ONE.

[32] Bashash M (2013). Genetic polymorphisms at TIMP3 are associated with survival of adenocarcinoma of the gastroesophageal junction. PLoS ONE.

[33] Bharthuar A (2014). Breast cancer resistance protein (BCRP) and excision repair cross complement-1 (ERCC1) expression in esophageal cancers and response to cisplatin and irinotecan based chemotherapy. J.

[34] Bradbury PA (2009). Cisplatin pharmacogenetics, DNA repair polymorphisms, and esophageal cancer outcomes. Pharmacogenet Genomics.

[35] Cescon DW (2009). p53 Arg72Pro and MDM2 T309G polymorphisms, histology, and esophageal cancer prognosis. Clinical Cancer Research.

[36] Huang Y, Peters CJ, Fitzgerald RC, Gjerset RA (2009). Progressive silencing of p14ARF in oesophageal adenocarcinoma. J Cell Mol Med.

[37] Renouf DJ (2013). Association of MDM2 T309G and p53 Arg72Pro polymorphisms and gastroesophageal reflux disease with survival in esophageal adenocarcinoma. Journal of gastroenterology and hepatology.

[38] Madani K, Zhao R, Lim HJ, Casson AG (2010). Prognostic value of p53 mutations in oesophageal adenocarcinoma: final results of a 15-year prospective study. European journal of cardio-thoracic surgery: official journal of the European Association for Cardio-thoracic Surgery.

[39] Loos M (2011). Clinical significance of the costimulatory molecule B7-H1 in Barrett carcinoma. The Annals of thoracic surgery.

[40] Fisher OM (2015). High Expression of Cathepsin E in Tissues but Not Blood of Patients with Barrett’s Esophagus and Adenocarcinoma. Ann Surg Oncol.

[41] Smith E (2016). Androgen Receptor and Androgen-Responsive Gene FKBP5 Are Independent Prognostic Indicators for Esophageal Adenocarcinoma. Dig Dis Sci.

[42] von Rahden BH (2010). Glucocorticoid-induced TNFR family-related receptor (GITR)-expression in tumor infiltrating leucocytes (TILs) is associated with the pathogenesis of esophageal adenocarcinomas with and without Barrett’s mucosa. Cancer biomarkers: section A of Disease markers.

[43] Borg D (2016). Expression of IFITM1 as a prognostic biomarker in resected gastric and esophageal adenocarcinoma. Biomark Res.

[44] Nguyen GH (2010). Inflammatory and microRNA gene expression as prognostic classifier of Barrett’s-associated esophageal adenocarcinoma. Clinical cancer research: an official journal of the American Association for Cancer Research.

[45] Derks S (2015). Epithelial PD-L2 Expression Marks Barrett’s Esophagus and Esophageal Adenocarcinoma. Cancer Immunol Res.

[46] Hu P (2015). Tumor-infiltrating neutrophils predict poor outcome in adenocarcinoma of the esophagogastric junction. Tumour Biol.

[47] Betts G (2014). FGFR2, HER2 and cMet in gastric adenocarcinoma: detection, prognostic significance and assessment of downstream pathway activation. Virchows Archiv: an international journal of pathology.

[48] Chan E (2016). EGFR family and cMet expression profiles and prognostic significance in esophagogastric adenocarcinoma. J.

[49] Mesteri I, Schoppmann SF, Preusser M, Birner P (2014). Overexpression of CMET is associated with signal transducer and activator of transcription 3 activation and diminished prognosis in oesophageal adenocarcinoma but not in squamous cell carcinoma. Eur J Cancer.

[50] Fisher OM (2015). MIC-1/GDF15 in Barrett’s oesophagus and oesophageal adenocarcinoma. British Journal of Cancer.

[51] Hu Y (2011). Prognostic significance of differentially expressed miRNAs in esophageal cancer. Int J Cancer.

[52] Izzo JG (2007). Clinical biology of esophageal adenocarcinoma after surgery is influenced by nuclear factor-kappaB expression. Cancer Epidemiol Biomarkers Prev.

[53] Mirza A (2014). Investigation of the epithelial to mesenchymal transition markers S100A4, vimentin and Snail1 in gastroesophageal junction tumors. Dis Esophagus.

[54] Singhi AD (2015). Smad4 loss in esophageal adenocarcinoma is associated with an increased propensity for disease recurrence and poor survival. Am J Surg Pathol.

[55] Howard JM (2014). Leptin and adiponectin receptor expression in oesophageal cancer. Br J Surg.

[56] Langer R, Feith M, Siewert JR, Wester HJ, Hoefler H (2008). Expression and clinical significance of glucose regulated proteins GRP78 (BiP) and GRP94 (GP96) in human adenocarcinomas of the esophagus. BMC Cancer.

[57] El-Mashed S (2015). LC3B globular structures correlate with survival in esophageal adenocarcinoma. BMC Cancer.

[58] Vashist YK (2014). EGFR intron-1 CA repeat polymorphism is a predictor of relapse and survival in complete resected only surgically treated esophageal cancer. Target.

[59] Izzo JG (2007). Cyclin D1 guanine/adenine 870 polymorphism with altered protein expression is associated with genomic instability and aggressive clinical biology of esophageal adenocarcinoma. Journal of clinical oncology: official journal of the American Society of Clinical Oncology.

[60] Aichler M (2014). Epidermal growth factor receptor (EGFR) is an independent adverse prognostic factor in esophageal adenocarcinoma patients treated with cisplatin-based neoadjuvant chemotherapy. Oncotarget.

[61] Dahle-Smith A (2015). Epidermal Growth Factor (EGFR) copy number aberrations in esophageal and gastro-esophageal junctional carcinoma. Mol Cytogenet.

[62] Langer R (2006). Prognostic significance of expression patterns of c-erbB-2, p53, p16 INK4A, p27KIP1, cyclin D1 and epidermal growth factor receptor in oesophageal adenocarcinoma: A tissue microarray study. Journal of clinical pathology.

[63] Ong CA (2013). Three-gene immunohistochemical panel adds to clinical staging algorithms to predict prognosis for patients with esophageal adenocarcinoma. Journal of clinical oncology: official journal of the American Society of Clinical Oncology.

[64] Wang KL (2007). Expression of epidermal growth factor receptor in esophageal and esophagogastric junction adenocarcinomas: association with poor outcome.[Erratum appears in Cancer. 2009 May 1;115(9):2024 Note: Reseetkova, Erika [corrected to Resetkova, Erika]]. Cancer.

[65] Hildebrandt MA (2009). Genetic variations in the PI3K/PTEN/AKT/mTOR pathway are associated with clinical outcomes in esophageal cancer patients treated with chemoradiotherapy. Journal of Clinical Oncology.

[66] Pavlov K (2015). GATA6 expression in Barrett’s oesophagus and oesophageal adenocarcinoma. Dig Liver Dis.

[67] Chan E (2016). Discordant HER2 expression and response to neoadjuvant chemoradiotherapy in esophagogastric adenocarcinoma. J.

[68] Langer R (2011). Assessment of ErbB2 (Her2) in oesophageal adenocarcinomas: summary of a revised immunohistochemical evaluation system, bright field double *in situ* hybridisation and fluorescence *in situ* hybridisation. Mod Pathol.

[69] Nagaraja V, Shaw N, Morey AL, Cox MR, Eslick GD (2016). HER2 expression in oesophageal carcinoma and Barrett’s oesophagus associated adenocarcinoma: An Australian study. Eur J Surg Oncol.

[70] Phillips BE (2013). Clinicopathologic features and treatment outcomes of patients with human epidermal growth factor receptor 2-positive adenocarcinoma of the esophagus and gastroesophageal junction. Dis Esophagus.

[71] Prins MJ, Ruurda JP, van Diest PJ, van Hillegersberg R, Ten Kate FJ (2013). The significance of the HER-2 status in esophageal adenocarcinoma for survival: an immunohistochemical and an *in situ* hybridization study. Ann Oncol.

[72] Prins MJ, Ruurda JP, van Diest PJ, van Hillegersberg R, ten Kate FJ (2014). Evaluation of the HER2 amplification status in oesophageal adenocarcinoma by conventional and automated FISH: a tissue microarray study. Journal of clinical pathology.

[73] Rauser S (2007). Significance of HER2 low-level copy gain in Barrett’s cancer: implications for fluorescence *in situ* hybridization testing in tissues. Clinical cancer research: an official journal of the American Association for Cancer Research.

[74] Slotta-Huspenina J, Becker KF, Feith M, Walch A, Langer R (2014). Heat Shock Protein 90 (HSP90) and Her2 in Adenocarcinomas of the Esophagus. Cancers (Basel).

[75] Yoon HH (2012). Association of HER2/ErbB2 expression and gene amplification with pathologic features and prognosis in esophageal adenocarcinomas. Clinical Cancer Research.

[76] Yoon HH (2012). Adverse prognostic impact of intratumor heterogeneous HER2 gene amplification in patients with esophageal adenocarcinoma. Journal of Clinical Oncology.

[77] Donohoe CL (2012). Role of the insulin-like growth factor 1 axis and visceral adiposity in oesophageal adenocarcinoma. Br J Surg.

[78] Smith E, Ruszkiewicz AR, Jamieson GG, Drew PA (2014). IGFBP7 is associated with poor prognosis in oesophageal adenocarcinoma and is regulated by promoter DNA methylation. British Journal of Cancer.

[79] Prins MJ, Verhage RJ, Ruurda JP, ten Kate FJ, van Hillegersberg R (2013). Over-expression of phosphorylated mammalian target of rapamycin is associated with poor survival in oesophageal adenocarcinoma: a tissue microarray study. Journal of clinical pathology.

[80] Bettstetter M (2013). Epidermal growth factor receptor, phosphatidylinositol-3-kinase catalytic subunit/PTEN, and KRAS/NRAS/BRAF in primary resected esophageal adenocarcinomas: loss of PTEN is associated with worse clinical outcome. Human pathology.

[81] Honing J (2014). Loss of CD44 and SOX2 expression is correlated with a poor prognosis in esophageal adenocarcinoma patients. Ann Surg Oncol.

[82] Sun L (2014). Prognostic impact of TAZ and beta-catenin expression in adenocarcinoma of the esophagogastric junction. Diagn Pathol.

[83] Honing J (2015). CD44, SHH and SOX2 as novel biomarkers in esophageal cancer patients treated with neoadjuvant chemoradiotherapy. Radiother Oncol.

[84] Lee JM (2011). Genetic variants in DNA repair predicts the survival of patients with esophageal cancer. Ann Surg.

[85] Becker L, Huang Q, Mashimo H (2010). Lgr5, an intestinal stem cell marker, is abnormally expressed in Barrett’s esophagus and esophageal adenocarcinoma. Dis Esophagus.

[86] von Rahden BH (2011). LgR5 expression and cancer stem cell hypothesis: clue to define the true origin of esophageal adenocarcinomas with and without Barrett’s esophagus?. Journal of experimental & clinical cancer research: CR.

[87] Lui, N. S., van Zante, A., Rosen, S. D., Jablons, D. M. & Lemjabbar-Alaoui, H. SULF2 expression by immunohistochemistry and overall survival in oesophageal cancer: a cohort study. *BMJ Open* **2**, 10.1136/bmjopen-2012-001624 (2012). 10.1136/bmjopen-2012-001624 PMC3532995 23180455

[88] Zhai R (2015). Whole-miRNome profiling identifies prognostic serum miRNAs in esophageal adenocarcinoma: the influence of Helicobacter pylori infection status. Carcinogenesis.

[89] Goh XY (2011). Integrative analysis of array-comparative genomic hybridisation and matched gene expression profiling data reveals novel genes with prognostic significance in oesophageal adenocarcinoma. Gut.

[90] Obulkasim A (2016). Reduced genomic tumor heterogeneity after neoadjuvant chemotherapy is related to favorable outcome in patients with esophageal adenocarcinoma. Oncotarget.

[91] Pennathur, A. *et al*. Gene expression profiles in esophageal adenocarcinoma predict survival after resection. *J Thorac Cardiovasc Surg* **145**, 505-512; discussion 512-503, 10.1016/j.jtcvs.2012.10.031 (2013). 10.1016/j.jtcvs.2012.10.031 PMC13268591 23321130

[92] Maru DM (2009). Frequent loss of heterozygosity of chromosome 1q in esophageal adenocarcinoma: loss of chromosome 1q21.3 is associated with shorter overall survival. Cancer.

[93] Davison JM (2014). The degree of segmental aneuploidy measured by total copy number abnormalities predicts survival and recurrence in superficial gastroesophageal adenocarcinoma. PLoS ONE.

[94] Dong H (2014). Snail1 correlates with patient outcomes in E-cadherin-preserved gastroesophageal junction adenocarcinoma. Clinical & translational oncology: official publication of the Federation of Spanish Oncology Societies and of the National Cancer Institute of Mexico.

[95] Slotta-Huspenina J (2012). Evidence of prognostic relevant expression profiles of heat-shock proteins and glucose-regulated proteins in oesophageal adenocarcinomas. PLoS ONE.

[96] Hofmann M (2008). Assessment of a HER2 scoring system for gastric cancer: results from a validation study. Histopathology.

[97] Myint ZW, Goel G (2017). Role of modern immunotherapy in gastrointestinal malignancies: a review of current clinical progress. Journal of Hematology & Oncology.

[98] Lee J, Tran P, Klempner S (2016). Targeting the MET Pathway in Gastric and Oesophageal Cancers: Refining the Optimal Approach. Clinical Oncology.

[99] Li H (2017). IGF-IR signaling in epithelial to mesenchymal transition and targeting IGF-IR therapy: overview and new insights. Molecular cancer.

[100] Lynce, F., Shajahan-Haq, A. N. & Swain, S. M. CDK4/6 inhibitors in breast cancer therapy: Current practice and future opportunities. *Pharmacol Ther*, 10.1016/j.pharmthera.2018.06.008 (2018). 10.1016/j.pharmthera.2018.06.008 PMC6533626 29933034

[101] Altorki N (2004). COX-2: a target for prevention and treatment of esophageal cancer. J Surg Res.

[102] Semenova G, Chernoff J (2017). Targeting PAK1. Biochem Soc Trans.

[103] Creemers, A. *et al*. Discordance in HER2 Status in Gastro-esophageal Adenocarcinomas: A Systematic Review and Meta-analysis. *Scientific Reports* **7** (2017). 10.1038/s41598-017-03304-9 PMC5466678 28600510

[104] Hanahan D, Weinberg RA (2011). Hallmarks of cancer: the next generation. cell.

