# Supplementary material for: A systematic review and meta-analysis of prognostic biomarkers in resectable esophageal adenocarcinomas
Source: Sci Rep. 2018 Sep 5;8:13281. doi: 10.1038/s41598-018-31548-6 (PMC6125467; doi:10.1038/s41598-018-31548-6)
Supplement: Supplementary file 1 — Supplementary Information S2-S5 [file 41598_2018_31548_MOESM1_ESM.pdf]

A systematic review and meta-analysis of prognostic biomarkers in resectable esophageal adenocarcinomas

Aafke Creemers[1, 2]*, Eva A. Ebbing[1, 2]*, Thomas C. Pelgrim[2], Sjoerd M. Lagarde[3], Faridi S. van Etten – Jamaludin[4] , Mark I. van Berge Henegouwen[5], Maarten C.C.M. Hulshof[6], Kausilia K. Krishnadath[1,7], Sybren L. Meijer[8], Maarten F. Bijlsma[1], Martijn G.H. van Oijen[2], Hanneke W.M. van Laarhoven[1,2]

[1]Laboratory of Experimental Oncology and Radiobiology, Amsterdam UMC, Univ of Amsterdam, Cancer Center Amsterdam, Amsterdam, The Netherlands [2] Department of Medical Oncology, Amsterdam UMC, Univ of Amsterdam, Cancer Center Amsterdam, Amsterdam, The Netherlands [3] Department of Surgery, Erasmus Medical Center, Rotterdam, The Netherlands [4] Department of Medical Library Science, Amsterdam UMC, Univ of Amsterdam, Cancer Center Amsterdam, Amsterdam, The Netherlands [5]Department of Surgery Amsterdam UMC, Univ of Amsterdam, Cancer Center Amsterdam, Amsterdam, The Netherlands [6]Department of Radiotherapy, Amsterdam UMC, Univ of Amsterdam, Cancer Center Amsterdam, Amsterdam, The Netherlands [7]Department of Gastroenterology, Amsterdam UMC, Univ of Amsterdam, Cancer Center Amsterdam, Amsterdam, The Netherlands [8]Department of Pathology, Amsterdam UMC, Univ of Amsterdam, Cancer Center Amsterdam, Amsterdam, The Netherlands

*These authors contributed equally to this work.

Supplementary table S2. Quality assessment of the included studies according to the adapted REMARK criteria.

Author		C1 cohort selection	C2 flow includions	C3 used material	C4 biomarker methods	C5 received therapy	C6 follow-up	C7 statistics	Total
Aichler, M.	2014	0,5	0,5	1	1	1	1	1	6
Bashash, M.	2013	1	1	1	1	1	1	1	7
Becker, L.	2010	1	1	1	1	0,5	1	0	5,5
Betts, G.	2014	0,5	1	1	1	1	1	1	6,5
Bettstetter, M.	2013	0,5	0,5	1	1	0	0	1	4
Bhandari, P.	2006	0,5	1	1	1	1	1	1	6,5
Bharthuar, A.	2014	0,5	1	1	1	1	1	0,5	6
Borg, D.	2016	1	1	1	1	1	1	1	7
Bradbury, P.A.	2009	0	0,5	0	1	1	1	1	4,5
Bradbury, P.A.	2009	0,5	1	1	1	0	1	1	5,5
Bradbury, P.A.	2009	1	1	1	1	1	1	1	7
Cescon, D	2009	0,5	1	0	1	0	1	1	4,5
Chan, E.	2016	1	1	1	1	1	1	0,5	6,5
Dahle-Smith, A.	2015	0,5	0,5	1	1	0	1	0,5	4,5
Davison, J.M.	2014	1	1	1	1	0	1	1	6
Davison, J.M.	2014	1	1	1	1	1	1	1	7
Derks, S.	2015	1	0	0	1	1	0	0,5	3,5
Dong, H.	2013	1	1	1	1	1	1	0,5	6,5
Dong, H.	2013	1	1	1	1	1	1	0,5	6,5
Dong, H.	2013	1	1	1	1	1	1	1	7
Donohoe, C.L.	2012	1	0,5	1	1	0	0	1	4,5
El-Mashed, S.	2015	0,5	0,5	1	1	1	0	1	5
Eng, L.	2015	1	1	1	1	0,5	1	1	6,5
Falkenback, D.	2008	1	1	1	1	1	0	0	5
Fisher, O.M.	2015	0,5	0	1	1	1	1	1	5,5
Fisher, O.M.	2015	0,5	0,5	1	1	1	1	1	6
Fisher, O.M.	2016	1	1	1	1	0,5	0	1	5,5
Goh, X.Y.	2011	0,5	0,5	1	1	0,5	?	0,5	4
Grimm, M.	2010	1	1	1	1	1	1	0,5	6,5
Hildebrandt, M.A.T.	2009	0,5	0,5	1	1	1	1	0,5	5,5
Honing, J.	2014	0,5	1	1	1	1	1	1	6,5
Honing, J.	2015	0,5	1	1	1	1	1	1	6,5
Howard, J.M.	2014	0,5	0,5	1	1	0	0	1	4
Hu, P.	2015	1	1	1	1	0,5	1	1	6,5
Hu, Y.	2011	0,5	0,5	1	1	1	0	1	5
Huang, J.	2009	0,5	0,5	1	1	1	0,5	1	5,5
Izzo, J.G.	2007	0,5	1	1	1	1	1	1	6,5
Izzo, J.G.	2007	0,5	1	1	1	1	1	1	6,5
Kim, S.M.	2010	0,5	0	1	1	0	0	1	3,5
Langer, R.	2006	1	1	1	1	1	1	0,5	6,5
Langer, R.	2008	0,5	0,5	1	1	1	1	0,5	5,5
Langer, R.	2011	0,5	1	1	1	1	1	1	6,5

Lee, M.J.	2011	0,5	1	1	1	1	0	1	5,5
Li, Z.	2015	1	1	1	1	1	1	1	7
Loos, M.	2011	0,5	0,5	1	1	1	1	0,5	5,5
Lu, X.	2016	1	1	1	1	0,5	1	1	6,5
Lui, N.S.	2015	1	1	1	1	0	1	1	6
Madani, K.	2010	0,5	1	1	1	1	1	0,5	6
Maru, D.M.	2009	1	0,5	1	1	0	1	0,5	5
Mesteri, I.	2014	0,5	1	1	1	0	1	1	5,5
Mirza, A.	2014	1	1	1	1	0,5	1	0,5	6
Nagaraja, V.	2016	1	1	1	1	0,5	0,5	0,5	5,5
Nguyen, G.H.	2010	0,5	1	1	1	1	1	1	6,5
Obulkasim, A.	2016	1	1	1	1	1	1	0	6
Ong, C.A.J.	2013	0,5	1	1	1	0	1	1	5,5
Pavlov, K.	2015	0,5	1	1	1	0	1	0,5	5
Pennathur, A.	2013	0,5	0,5	1	1	0	1	1	5
Phillips, B.E.	2013	1	1	1	1	1	1	1	7
Prins, M.J.	2012	0,5	1	1	1	0	1	1	5,5
Prins, M.J.	2013	0,5	1	1	1	1	1	1	6,5
Prins, M.J.	2015	0,5	1	1	1	1	1	1	6,5
Prins, M.J.	2014	0,5	1	1	1	0	1	1	5,5
Prins, M.J.D.	2013	0,5	1	1	1	1	1	1	6,5
Rauser, S.	2007	1	0	1	1	1	1	1	6
Renouf, D.J.	2013	0,5	1	0	1	1	1	1	5,5
Singhi, A.D.	2015	0,5	1	1	1	0	1	1	5,5
Slotta-Huspenia, J.	2012	0,5	1	1	1	1	1	1	6,5
Slotta-Huspenia, J.	2014	1	1	1	1	0	1	0	5
Smith, E.	2014	1	0,5	1	1	1	1	1	6,5
Smith, E.	2016	1	1	1	1	1	1	0	6
Streppel, M.M.	2012	0,5	0,5	1	1	0	0	1	4
Sun, L.	2014	1	1	1	1	1	1	1	7
Tokunaga, R.	2015	1	0,5	1	1	1	1	1	6,5
Underwood, T.J.	2015	0,5	0	1	1	0	1	0,5	4
Vashist, Y.K.	2012	1	1	1	1	1	1	0,5	6,5
Vashist, Y.K.	2014	0,5	0	1	1	0	0	1	3,5
von Rahden, B.H.A.	2010	0,5	1	1	1	0,5	1	0,5	5,5
von Rahden, B. H.A.	2011	1	1	1	1	1	1	0,5	6,5
Wang, K.L.	2006	1	1	1	1	0	1	1	6
Wang, K.L.	2007	1	1	1	1	0	1	1	6
Xie, L.X.	2013	1	1	1	1	1	1	1	7
Yoon, H.H.	2012	1	1	1	1	1	1	1	7
Yoon, H.H.	2012	1	1	1	1	1	1	1	7
Zhai, R.	2015	0,5	1	1	1	0,5	0	1	5

Supplementary figure S3.

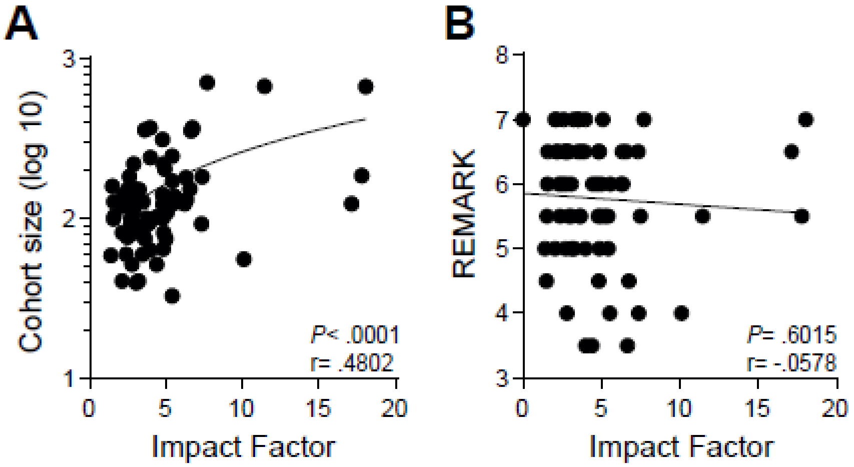

Supplementary figure S3. Correlations between cohort size and impact factor (A) and scored quality of the included articles on the adapted REMARK criteria scale and impact factor (B).

Supplementary figure S4.

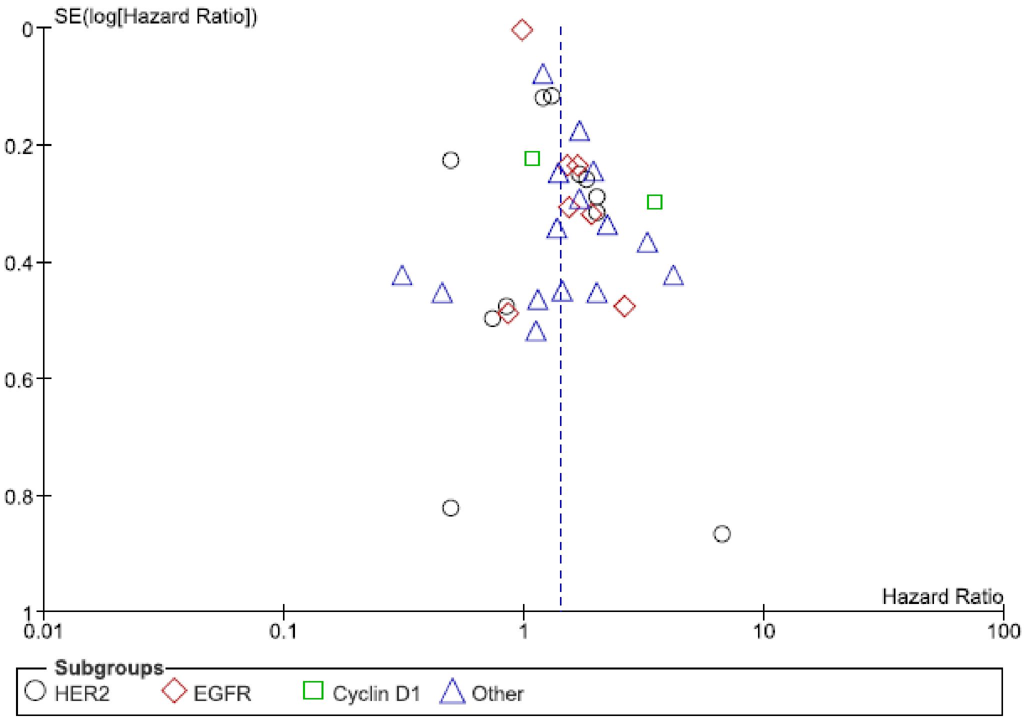

Supplementary figure S4. Funnel plot assessing publication bias of the adapted hallmark of cancer 'proliferation'.

Supplementary information S5.

Literature search conducted: 19-1-2017

Total 6494 hits
Duplicates removed total: 3294 hits.

MEDLINE (Ovid):
Database(s): Epub Ahead of Print, In-Process & Other Non-Indexed Citations, Ovid MEDLINE(R) Daily and Ovid MEDLINE(R) 1946 to Present
Search Strategy:

#	Searches	Results
1	(*Esophageal Neoplasms/ or Esophagectomy/ or ((esophagus or esophageal or esophagogastric or oesophagus or oesophageal or oesophagogastric or gastroesophag* or gastrooesophag*) adj3 (neoplas* or cancer* or carcino* or adenocarcino* or tumor* or tumour* or malig*)).ti,ab. or (esophagectom* or oesophagectom*).ti,ab.) and (survival or prognos*).ti,ab.	14620
2	Survival/ or exp Survival Analysis/ or Survival Rate/ or Prognosis/ or (surviv* or kaplan-meier or prognos* or predict* or longterm or long-term).ti,ab.	3323917
3	exp Biological Markers/ or Gene Expression/ or "Gene Expression Regulation, Neoplastic"/ or Gene Expression Profiling/ or Esophageal Neoplasms/ge or (biomarker* or marker* or expressi* or expressed or overexpressi* or overexpressed).ti,ab.	3511265
4	1 and 2 and 3	4532
5	limit 4 to (english and last 10 years)	3245

EMBASE (Ovid):
Database(s): Embase Classic+Embase 1947 to 2017 January 18
Search Strategy:

#	Searches	Results
1	(exp *esophagus tumor/ or *esophagus resection/ or ((esophagus or esophageal or esophagogastric or oesophagus or oesophageal or oesophagogastric or gastroesophag* or gastrooesophag*) adj3 (neoplas* or cancer* or carcino* or adenocarcino* or tumor* or tumour* or malig*)).ti,ab. or (esophagectom* or oesophagectom*).ti,ab.) and (survival or prognos*).ti,ab.	19090
2	exp survival/ or Kaplan Meier method/ or exp prognosis/ or (surviv* or kaplan-meier or prognos* or predict* or longterm or long-term).ti,ab.	3980256
3	exp marker/ or gene expression/ or gene overexpression/ or gene expression regulation/ or gene expression profiling/ or genotype/ or gene amplification/ or (biomarker* or marker* or expressi* or expressed or overexpressi* or overexpressed).ti,ab.	4334235
4	1 and 2 and 3	5980
5	(animal*.hw. or exp animal/) not human/	5432962
6	4 not 5	5905
7	limit 6 to (english and last 10 years)	4559
8	limit 6 to (conference abstract or conference paper or conference proceeding or "conference review")	1477
9	7 not 8	3105

Cochrane Central Register of Controlled Trials
http://onlinelibrary.wiley.com/cochranelibrary/search/advanced/shared/searches/6515280765701507567
166 trials
ID Search Hits
#1 ((esophagus or esophageal or esophagogastric or oesophagus or oesophageal or oesophagogastric or gastroesophag* or gastrooesophag*) near/3 (neoplas* or cancer* or carcino* or adenocarcino* or tumor* or tumour* or malig*)):ti,ab,kw or (esophagectom* or oesophagectom*):ti,ab,kw 2823
#2 (surviv* or kaplan-meier or prognos* or predict* or longterm or long-term):ti,ab,kw 168115
#3 (biomarker* or marker* or expressi* or expressed or overexpressi* or overexpressed or gene* or genotype*):ti,ab,kw 159342
#4 MeSH descriptor: [Gene Expression Regulation] explode all trees 2306
#5 #3 or #4 159836
#6 #1 and #2 and #5 Publication Year from 2007 to 2017, in Trials 166
